# Urban Water Access and Use in the Kivus: Evaluating Behavioural Outcomes Following an Integrated WASH Intervention in Goma and Bukavu, Democratic Republic of Congo

**DOI:** 10.3390/ijerph19031065

**Published:** 2022-01-18

**Authors:** Hugo Legge, Shahana Fedele, Florian Preusser, Patrycja Stys, Papy Muzuri, Moritz Schuberth, Robert Dreibelbis

**Affiliations:** 1 Department of Disease Control, Faculty of Infectious and Tropical Diseases, London School of Hygiene and Tropical Medicine, London WC1E 7HT, UK; Robert.Dreibelbis@lshtm.ac.uk; 2 Mercy Corps, Edinburgh EH6 6LX, UK; shahanafedele@hotmail.com (S.F.); florianpreusser@googlemail.com (F.P.); moritz.schuberth@gmail.com (M.S.); 3 Firoz Lalji Institute for Africa, London School of Economics and Political Science, London WC2A 2AE, UK; p.m.stys@lse.ac.uk (P.S.); muzuribat@gmail.com (P.M.)

**Keywords:** water supply, water use, urban, hygiene, behaviour

## Abstract

Increasing the availability and reliability of community water sources is a primary pathway through which many water supply interventions aim to achieve health gains in communities with limited access to water. While previous studies in rural settings have shown that greater access to water is associated both with increased overall consumption of water and use of water for hygiene related activities, there is limited evidence from urban environments. Using data collected from 1253 households during the evaluation of a community water supply governance and hygiene promotion intervention in the cities of Goma and Bukavu, Democratic Republic of Congo, we conducted a secondary analysis to determine the impact of these interventions on household water collection and use habits. Using multiple and logistic regression models we compared differences in outcomes of interest between households in quartiers with and without the intervention. Outcomes of interest included litres per capita day (lpcd) of water brought to the household, lpcd used at the household, and lpcd used for hygiene-related activities. Results demonstrated that intervention households were more likely to use community tapstands than households located in comparison quartiers and collected on average 16.3 lpcd of water, compared with 13.5 lpcd among comparison households (adj. coef: 3.2, 95 CI: 0.84 to 5.53, *p* = 0.008). However, reported usage of water in the household for domestic purposes was lower among intervention households (8.2 lpcd) when compared with comparison households (9.4 lpcd) (adj. coef: −1.11, 95 CI: −2.29 to 0.07), *p* = 0.066) and there was no difference between study groups in the amount of water allocated to hygiene activities. These results show that in this setting, implementation of a water supply governance and hygiene promotion intervention was associated with a modest increase in the amount of water being bought to the household, but that this did not translate into an increase in either overall per capita consumption of water or the per capita amount of water being allocated to hygiene related activities.

## 1. Introduction

Increasing access to basic and safely managed drinking water is a global target that is enshrined in Sustainable Development Goal 6. Significant gains have been made across the globe over past decades in improving access to safe water supplies. However, in Sub-Saharan Africa over a third of households still rely on limited or unimproved sources [1].

Water supply interventions in communities with limited access to improved water sources often target both improvements in water availability and—when coupled with source protection or treatment—improvements in water quality [2]. Increasing the availability of drinking water at either the household, community, or both levels has been associated with both increased water consumption at the household level as well as a subsequent increase in the allocation of water resources to hygiene activities [3,4,5]. However, the majority of research investigating the impact of hygiene promotion and water supply interventions has so far been limited to rural settings and the extent to which findings are transferrable to an urban environment is unknown [6,7]. With urbanization accelerating across the globe, and in particular in Sub-Saharan Africa, there is a clear need to address this gap in the literature [8].

In 2008, Mercy Corps (MC) and USAID launched the first phase of a project to rehabilitate and extend the municipal water system of the city of Goma in the Democratic Republic of Congo, which was degraded by decades of mismanagement, and partially destroyed by lava flows from the 2002 volcanic eruption of Mount Nyiragongo. In 2013, MC signed a grant agreement with the UK Department for International Development (DFID) for the Integrated Maji Infrastructure and Governance Initiative for Eastern Congo (IMAGINE) to scale-up the Goma water project and extend it to the city of Bukavu. In 2015, MC commenced a behaviour change communication (BCC) component to improve hygiene behaviours and water management practices at both the household and communal level, including handwashing and safe water storage and treatment.

As part of their 2019 endline evaluation, MC collected detailed data on households’ water collection and use habits. These data provide an opportunity for a secondary analysis with the objective of investigating how improvements in water source governance and delivery of a hygiene BCC intervention in an urban setting translate into changes in water collection and use at the household level. Specifically, this analysis seeks to assess if regulating tapstand operating hours and standardizing pricing of community water sources in the urban environment of Goma results in an increase in household water collection and use. In parallel to this, we assess whether the implementation of a hygiene behaviour change campaign results in an increase in water usage at the household level in selected quartiers of the city of Bukavu. This analysis is the first to quantify unmetered household water consumption in DRC and will be of relevance to researchers and implementers working in water supply and hygiene promotion in the DRC and other urban settings where access to private household water connections is limited [9].

## 2. Methods

### 2.1. Study Setting and Intervention Summary

**Figure 1:**
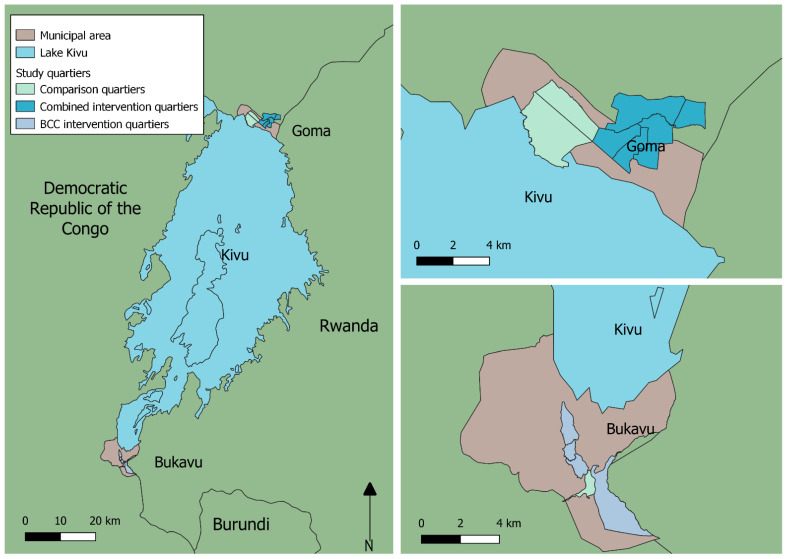
The study sites and surrounding area.

The city of Goma, the capital of the province of North Kivu, is located in the east of the Democratic Republic of Congo (DRC) on the shores of Lake Kivu, and on the border with Rwanda (Figure 1). The city lies south of the active Nyiragongo volcano and is dominated by a tropical, rainy, and dry climate, with annual temperatures of 19.9 °C and an annual rainfall of 1192 mm. Volcanic eruptions of Mount Nyiragongo and protracted armed conflicts have had consequences on the city and its surroundings, particularly on the resettlement of the population [10,11]. Given its strategic location as a regional commercial centre, Goma’s population has grown significantly in the last decades as flow of people were attracted by the new labour market as well as by the construction boom which benefits from the money generated by the mineral trade [12].

The city of Bukavu is the administrative and commercial centre of the South Kivu Province and extends south from the shore of Lake Kivu and along the Ruzizi River, whilst bordering with Rwanda. The topography of the city is characterised by deep valleys and steep hills, on which much of the city is built. The climate is tropical with small temperature variations. The average daily temperature is very similar to Goma’s and the average total annual rainfall is 1391 mm, with significant variations according to the seasons. Bukavu is characterised by a high density of growing population, a poor socio-economic situation, and a lack of infrastructure and basic services [13]. Commerce is the main economic activity of the city and trade activities are also carried out with neighbouring Rwanda.

Since 2008, MC’s efforts to improve the delivery of water services in eastern DRC have included infrastructure improvements coupled with management reform, with the objective to provide a sustainable long-term management solution for infrastructure assets. After having put in place a pilot management system for the existing 52 public tap stands connected to the water network north of Goma between 2014 and 2016, and following a comprehensive water utility contracting and operating study carried out to design the optimum dispositions for the delegated management of the network, MC decided to facilitate the creation of a DRC water utility company called Congo Maji Sarl. This company signed a public private partnership contract with the state-owned water company REGIDESO for the management of water utilities for parts of the city of Goma in August 2018. This management system resulted in standardised pricing and regular operating hours across the 52 existing public tap stands.

In addition to this, between 2015 and 2019, MC delivered hygiene-related messaging to residents of both Goma and Bukavu through local radio, television, street theatre, and street cinema. In addition to this, MC established *care groups* in collaboration with local health authorities, which saw local volunteers trained as community-based behaviour change agents, tasked with disseminating and promoting BCC messaging. Specific topics covered included handwashing, food hygiene, sanitation, exclusive breastfeeding, diarrhoea prevention, safe management, cholera prevention, use of oral rehydration salts with the overall objective to improve hygiene behaviours, and water management practices at both the household and communal level, including handwashing and safe water storage and treatment. In total, 472 care groups were established across Goma and Bukavu with 6576 volunteers trained.

### 2.2. Study Design

In Goma, data were collected from households in five quartiers (the administrative unit below a commune) that received the BCC and water governance interventions (*n* = 283) and from households from two neighbouring quartiers that received neither (*n* = 356). In Bukavu, data were collected from households in quartiers where the BCC intervention was delivered (*n* = 302) and households from neighbouring quartiers where no intervention was delivered (*n* = 312). No randomisation occurred as part of this study, with intervention quartiers selected based on logistical considerations and comparison quartiers selected in order to maximise geographic proximity and sociodemographic comparability with intervention quartiers. A one-stage stratified sampling methodology was used to randomly sample 1253 households from two strata (Goma, *n* = 639; Bukavu, *n* = 614) over a 19-day data collection period in May and June 2019. Surveys were administered to adults in each household following provision of informed consent by an adult household member. Ethical approval for this secondary analysis was provided by the London School of Hygiene and Tropical Medicine ethics committee (19107). Surveys were conducted where water collection habits during the previous day were self-reported by the respondent and water usage habits were demonstrated using an interactive survey tool which asked the respondent to indicate water usage over the previous day using containers available in the household as visual aids.

### 2.3. Outcomes

Previous studies have used the amount of water bought to the house over a given time period as a proxy indicator for domestic water consumption [6]. However, water collection and water use are conceptually distinct activities that involve separate processes. As a result, these measures may present different quantities if, for example, water is collected or purchased on one day and shared for usage among many households or conversely stored and used over an extended time period. For this reason, the two were measured separately in this study. We defined water collection as the act of drawing and transporting water from a water source off or on the household compound by a household or non-household member for the purpose of either storage or immediate use. Water use was defined as water used inside the household premises by any household members for non-commercial use.

The BCC intervention (delivered in Bukavu) and the combined water governance and BCC intervention (delivered in Goma) were both hypothesised as having potential positive impacts on water usage at the household level. As a result, the total amount of water consumed at the household level and the total amount used for hygiene related activities, such as handwashing, were assessed as outcomes of interest in analyses for both study sites. Additional indicators relating to water collection, time spent collecting water by household members, and the associated costs of drawing water were hypothesised as being impacted by the water governance component of the intervention delivered in Goma. As a result, these outcomes were assessed as part of the analysis for the Goma study site only.

Water collection: Litres per capita per day (lpcd) of water collected the previous day was the primary water collection outcome included in our analysis. This was calculated using amount of water collected from sources off the compound (e.g., surface water) or from within the compound (e.g., private stand pipe or rainwater harvesting) by individuals inside or outside of the household (e.g., bicycle vendors). A binary indicator was established to measure whether the primary respondent reported any water collection taking place during the previous day. If water was collected, the amount was calculated by asking the primary respondent to indicate what source types they had visited during the previous day or had delivered to their household, and then asking them to recall the container types (5/10/20 L container) that had been used and the frequency with which each container type had been filled. The total amount collected from all water sources collected during the previous day was then calculated and divided by the number of household members to generate the per capita amount collected yesterday. This can be observed in Equations (1) and (2):

(1) $$Amount\;collected\;from\;a\;given\;source\;type\;yesterday=\overset{3}{\underset{i=1}{\sum }}x_{i}y_{i}$$

where x indicates container type and y indicates frequency of being filled

(2) $$Per\;capita\;amount\;of\;water\;collected\;yesterday=\frac{\;\sum_{j=1}^{11}S_{j}}{h}$$

where *j* represents different source types, *S* is the amount collected from a water source, and *h* is the number of household members.

Average daily per capita collection of water was calculated at the study group level by multiplying the proportion of households that collected any water yesterday (a measure of the likelihood of collecting water on a given day) by the mean average per capita amount collected yesterday among households that did collect water.

Eleven different water source types were identified as being in regular use in the study communities (Table 1). These were additionally classified into current JMP drinking water ladder categories as part of the analysis [14]. Due to a lack of reliable data on water quality it was not possible to classify any of the sources as “safely managed”. Instead, we classified “Piped on premises” as an alternative top-tier category which meets the majority of the requirements for “safely managed”. Rainwater and dug wells, while normally classified as improved sources, were deemed to be unimproved due to these sources commonly being unprotected in both sites.

*Time spent collecting*: Time spent collecting water at the household level was measured by two outcomes. The first was the daily per capita number of minutes the primary respondent reported that a household member spent collecting water. Time spent was estimated by combining self-reported time to travel to the source combined with the reported queuing and drawing time and multiplied by the frequency of trips to the source (based on observations carried out during piloting it was estimated that on average an individual carried 20 L worth of water per trip). This can be observed in Equation (3).

(3) $$Time\;spent\;collecting\;water\;yesterday=\overset{11}{\underset{i=1}{\sum }}\left(\frac{S_{i}}{20}\right)2d_{i}+q_{i}$$

where S = amount of water in litres collected yesterday from a source, d = time taken to travel to the water source, and q = the queuing and drawing time for the water source.

The second outcome aimed to measure the efficiency of water collection through establishing the number of litres collected per minute spent collecting. This was calculated by dividing the total amount collected yesterday by the total time spent collecting yesterday.

*Water source heterogeneity*: Within-household source heterogeneity was measured by self-reported number of sources accessed in general in addition to proportion of water collected during the previous day that came from the household’s primary source.

*Costs of water collection*: The cost of water collection was measured by asking respondents to self-report the method of payment (i.e., per container or regular scheduled payment) and associated costs. This was then scaled to the amount that was collected during the previous day to generate the daily total cost of water in addition to the average cost per litre.

*Water usage*: The specific indicator employed to measure water usage was per capita usage for domestic purposes within the household compound during the previous day. The usage categories used in the survey were bathing, washing hands, drinking, cooking, cleaning, sanitation, and laundry. Water that was reused for a secondary activity after being initially used was also included as part of this measurement.

### 2.4. Analysis

Statistical analysis of this data was undertaken using Stata15 (Stata Corp, College Station, TX, USA) and graphics were generated using the ggplot2 package in R. Following data assembly and cleaning, sociodemographic characteristics of the study sites were tabulated and summarised by study group. Due to the absence of randomisation in the study design and the hypothesised differences in socioeconomic status (SES) and the availability of water sources between quartiers, all models were controlled for SES and self-reported travel-time to the main water source. To create an SES score, an iterative principal component analysis (PCA) of binary asset and household utility variables was completed. Items were retained in the final PCA if they had an item-rest correlation of >0.1 and the PCA was considered to have acceptable levels of internal consistency when the overall alpha was >0.7 [15]. Categorical versions of these variables based on quintiles were then included in all outcome models to control for differences in socioeconomic status between households. For continuous outcome variables, multiple linear regression models were used to generate adjusted coefficients to quantify differences between study groups. For binary outcome variables, multiple logistic regression was performed to output adjusted odds ratios. For both continuous and binary outcome variables a *p*-value < 0.05 was used as the significance threshold.

## 3. Results

### 3.1. Goma

Demographic variables were broadly similar between all three study groups, however, households in the comparison group had marginally higher representation in the two highest socioeconomic score quintiles (Table 2).

### 3.2. Water Collection, Time Spent Collecting, and Costs

Furthermore, 81% of households receiving the combined intervention collected water during the previous day compared with 63% of households in the comparison quartiers (Table 3). After adjusting for SES and distance to main water source, households in intervention quartiers had 2.5 times the odds of collecting any water (including unimproved sources) during the previous day compared with comparison households (95% confidence interval (CI): 1.76–3.65). Among households that collected any water during the previous day there was no significant difference in the reported amount collected between comparison (19.58 lpcd) and intervention (19.92 lpcd) households. However, among all households, including those that did not collect water during the previous day, intervention households collected significantly more water (16.33 lpcd) than comparison households (13.53 lpcd) (adj. coef: 3.19, 95 CI: 0.84–5.53, *p* < 0.001).

Among households that collected water during the previous day, comparison households spent 13.1 min per capita day collecting water compared with 17.8 min per capita day among intervention households (adj. coef: 4.46, 95 CI: 1.98–6.93, *p* < 0.001). The efficiency of time spent collecting water from any source was higher among households in comparison quartiers, who collected on average 3.1 L of water per minute spent collecting, when compared with intervention households, even after adjusting for distance to main water source (adj. coef: −0.1, 95 CI: −1.2 to −0.7, *p* < 0.001). When comparing efficiency in collection time among households collecting water from tapstands, households in intervention quartiers were more efficient (1.2 L per minute compared with 0.7 L per minute. However, this difference was not significant after adjusting for distance to water source and SES (adj. coef: 0.1, 95 CI: −0.3 to 0.5, *p* > 0.1).

There was no significant difference between study groups in the overall cost per litre of water collected, with comparison and intervention households paying on average CDF 4.19 /L and CDF 4.41/L respectively (adj. coef: 0.29, 95 CI: −0.95 to 1.53, *p* > 0.1). However, the cost of water from improved and piped to plot sources was on average CDF 0.6/L more expensive in comparison quartiers when compared with intervention quartiers (95 CI: −1.09 to −0.13, *p* = 0.014).

### 3.3. Water Source Heterogeneity

**Figure 2:**
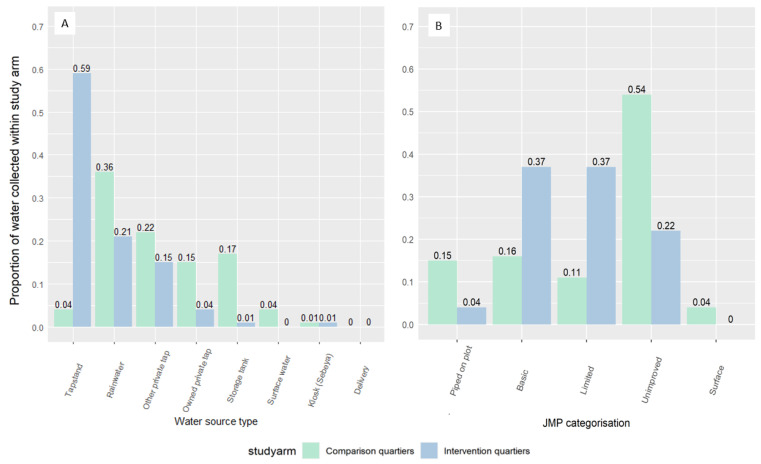
Proportion of water collected during the previous day in Goma by study group and (**A**) water source type, and (**B**) JMP water source type. NB, no water was collected from unprotected dug wells, shallow wells, and unprotected springs.

There were relatively high levels of homogeneity in water source accessed among households in intervention quartiers; 59% of the water collected during the previous day was reported to come from public tapstands (Figure 2). In comparison quartiers, water source heterogeneity was greater. Rainwater harvesting systems accounted for the majority of water sources used but provided only 36% of all water accessed during the previous day. Among comparison households, tapstands were not widely accessed and accounted for only 4% of water collected the day before data collection.

### 3.4. Water Usage

**Figure 3:**
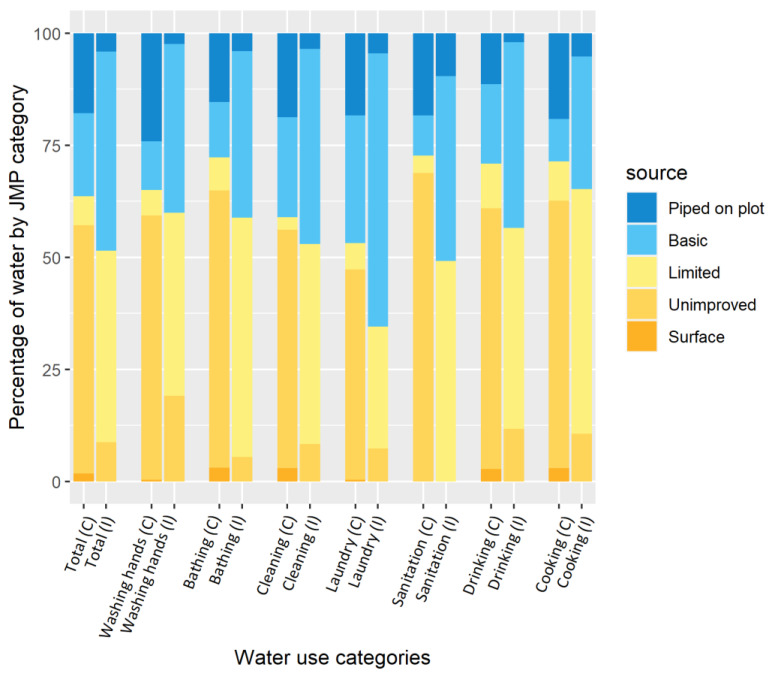
Proportion of water used during the previous day in Goma by JMP categories in I comparison and (I) combined intervention households.

Water usage (from any source) in the household was lower in intervention households (8.2 lpcd) compared with comparison households (9.4 lpcd) (adj. coef: −1.1, 95 CI: −2.3 to −0.1, *p* = 0.066) (Table 4). Intervention households used less water for household cleaning (adj. coef: −0.3, 95 CI: −0.5 to −0.1, *p* = 0.003). There was no difference between study groups in the amount of water allocated to handwashing, bathing, cooking, drinking, or laundry. Water drawn from unimproved sources was more widely used for all activities in comparison households when contrasted with intervention households (Figure 3).

### 3.5. Bukavu

While demographic variables were broadly similar between comparison and BCC intervention households there were appreciable differences in socioeconomic status (SES) with intervention households having larger representation in higher SES quintiles.

### 3.6. Water Usage

Households in the BCC intervention quartiers used on average 5.92 L of water per person per day compared with 5.28 L in the comparison quartier (adj. coef: 0.68, 95 CI: −0.31 to 1.66, *p* < 0.1) (Table 5). BCC households used fractionally more water for handwashing (0.09 lpcd), however, after adjustment for socioeconomic status and distance to water source this difference was not significant. There were no significant differences in water usage across any category.

## 4. Discussion

In quartiers in Goma where the combined water supply governance and behaviour change intervention was delivered, we observed a modest increase in the per-capita amount of water collected despite overall reported usage at the household being lower when contrasted with comparison households. These findings show that in the urban environment of Goma, rehabilitating community water sources and instituting regular operating hours and standardised pricing, in addition to implementing a sustained hygiene BCC intervention was not sufficient to increase either the overall amount of water used for domestic purposes nor the water allocated specifically to hygiene activities consumed at the household.

However, use of improved sources, specifically tapstands, was significantly higher in intervention quartiers suggesting that the water supply governance intervention may have resulted in households switching from unimproved to improved sources while not increasing the amount of water that they were using. In Bukavu, where only the BCC intervention was delivered, there was no evidence that the intervention had increased either the overall amount of water used for domestic purposes nor the amount used for hygiene related activities. These findings indicate that the BCC intervention alone was not sufficient to alter the amount of water being allocated to key hygiene behaviours promoted as part the BCC intervention.

While previous studies have shown that targeted, behaviour-orientated health promotion campaigns can result in improvements in observed hygiene practices and the availability of hygiene-related infrastructure and materials, few have examined whether these interventions increase the amount of water being consumed [16,17,18,19]. In this setting, results suggest that BCC interventions alone are unlikely to increase the amount of water being consumed for hygiene purposes in the household.

Previous studies have used *behaviour settings theory* to explore how household water consumption is shaped by both the physical and social environment in which it occurs [20,21]. From a behavioural settings perspective, interventions that fail to disrupt the settings in which routine behaviours occur—the unique constellation of infrastructure, materials, norms, competencies, and routines associated with a specific behavioural objective—are unlikely to result in changes to behaviours. In the context of this study, the rehabilitation and improved management of public tapstands in Goma may have changed the behaviour setting of water collection and resulted in households utilising improved water sources in greater numbers; however, the BCC intervention alone was not sufficient to disrupt the behaviour setting of water use within the household, and as a result only marginal changes in water use routines were observed. These results are consistent with previous research conducted in rural or peri-urban settings which have shown that increasing the availability of community water sources in areas with high levels of water source heterogeneity delivers only minor increases in household water consumption unless and until household water connections are established [3,4,22,23,24,25,26,27].

In both study groups in Goma, the average daily per-capita amount of water being collected and brought to the household was markedly higher than the daily per-capita amount reported as being used at the household. The discrepancy between these two estimations could be the result of water being brought to the household and used for commercial purposes, such as watering crops or livestock, or the redistribution of water among neighbouring households after its initial collection. These results highlight the conceptual distinction that should be drawn between water collection and water usage, as water collected and brought to the household does not necessarily equate to water consumed for domestic purposes. This finding supports conclusions from a recent systematic review of methodologies for measuring unmetered domestic water usage in low-income settings which suggested that household domestic water usage may be a more relevant indicator for hygiene practices and related human health outcomes than household water collection [6].

Results from the cost and time allocation analysis showed that the average cost per litre of water was similar among all households regardless of intervention status, while the cost per litre of water from improved sources was slightly cheaper in intervention quartiers. Water collection was less efficient in intervention quartiers, with more time expended per litre of water collected when compared with comparison quartiers. These findings may be explained by households in the intervention quartier transitioning away from easily accessible and lower cost unimproved sources, such as rainwater, towards fee-charging tapstands. That use of community tapstands was higher in intervention quartiers, despite the absence of any cost of time-saving benefit, suggests that users were prioritizing these sources based on other considerations. This finding supports evidence from previous studies in rural settings in Ghana, Nepal, and India, which have shown that prioritisation of water sources can rest on perceptions of taste, the reliability of the source in question, and the functional ease with which water can be withdrawn [28,29,30].

The lack of randomisation of quartiers into different study groups means that this study is vulnerable to confounding. Specifically, we cannot rule out the possibility that observed differences between study groups are due to pre-existing differences in water access and socio-demographic conditions in the study groups. Through controlling for socioeconomic status and distance to main water source within our models we have attempted to account for these differences, but some residual confounding may have persisted. Data were collected over a 19-day period in May and June 2019 and as a result do not capture seasonal variations in climate and water availability which are likely to impact water collection and usage habits [31]. Additionally, it is possible that a longer period between conclusion of the intervention and collection of the endline data would have resulted in a stronger link between intervention and outcome as WASH interventions have been shown to be time lagged in their impact [32]. Data was collected prior to MC undertaking further work to increase the number of tapstands available in Goma. Access to hygiene infrastructure and supplies, such as handwashing stations and presence of soap, were not collected as part of this survey, which prohibited analysis examining the relationship between access to hygiene and water usage associated with hygiene activities. Accurate data on the condition of individual water sources referenced within this paper was not available. As a result, a conservative approach to water source classification was adopted, which may have resulted in some water sources which meet the requirements of “improved” being classified as “unimproved”.

## 5. Conclusions

In this urban setting, a water supply governance intervention, delivered in hand with a targeted hygiene BCC, was successful in promoting access to and use of community tapstands within the city of Goma. Where tapstands had been rehabilitated, households prioritized their use despite the potential availability of cheaper and more easily accessible water from unimproved sources. However, the potential implications of this change on safer water consumption require further investigation as several studies have identified a rapid deterioration in water quality after source collection [33,34,35]. With regards to household consumption, the intervention was not sufficient to increase either the overall amount of water being consumed or the amount of water being allocated to key hygiene activities at the household level. Daily per capita consumption of water was well below the WHO benchmark of 50 lpcd in both study groups [36], providing evidence that water supply interventions in urban settings delivered at the community level may not be sufficient to drive household water usage up to the minimum recommended standards. Findings from this study suggest that WASH programmes aiming to increase household water consumption should be cautious about focusing exclusively on community level infrastructure. Instead, evidence from other studies in similar settings suggests programmes seeking an increase in consumption should consider addressing barriers to household water connections where feasible [9,35].

## Figures and Tables

**Table 1:** Water source types identified as being in use in the study communities.

Type	Source	Description
Piped on premises	Private tap (owned)	Private tapstand owned by accessing household
Basic or limited *	Tapstand	Public tapstand-including all pre-existing tapstands and those newly constructed or rehabilitated by the IMAGINE project.
	Private tap (other)	Private tapstand not owned by accessing household
Unimproved	Kiosk	Sebeya—Water sold at kiosks that is pre-bottled and transported from sources in Rwanda
	Storage tank	Water stored in large storage containers from various sources
	Shallow well	Bizola—A traditional unprotected shallow dug well
	Dug well	Unprotected dug well
	Unprotected spring	Unprotected spring
	Rainwater	Rainwater, typically harvested from within household compound
	Delivery	Water sold from bicycle vendors sourced from lake Kivu
Surface water	Surface water	Including water accessed directly from rivers and Lake Kivu

* Classified as basic if the source was less than a 30-min round trip from the household.

**Table 2:** Socio demographic and water access characteristics of households in Goma and Bukavu by study group.

	Bukavu (*n* = 614)		Goma (*n* = 639)	
	Comparison, *n* (%)	BCC Intervention, *n* (%)	Comparison, *n* (%)	Combined Intervention, *n* (%)
Wealth index quintile				
Lowest wealth quintile	109 (34.7)	24 (7.8)	63 (17.7)	61 (21.6)
2	72 (22.9)	38 (12.3)	62 (17.4)	59 (20.8)
3	74 (23.6)	44 (14.2)	66 (18.5)	67 (23.7)
4	40 (12.7)	97 (31.4)	91 (25.6)	62 (21.9)
Highest wealth quintile	19 (6.1)	106 (34.3)	74 (20.8)	34 (12)
Number of household members				
1–4	59 (18.8)	43 (13.9)	123 (34.6)	91 (32.2)
5–7	129 (41.1)	119 (38.5)	162 (45.5)	137 (48.4)
7+	126 (40.1)	147 (47.6)	71 (19.9)	55 (19.4)
Respondent level of education				
No education	50 (15.9)	25 (8.1)	16 (4.5)	14 (4.9)
Primary education	87 (27.7)	46 (14.9)	46 (12.9)	43 (15.2)
Secondary or above	177 (56.4)	238 (77)	294 (82.6)	226 (79.9)
Ownership of dwelling				
Own dwelling	206 (65.6)	212 (68.6)	230 (64.6)	169 (59.7)
Occupation of head of household				
Unemployed	25 (8.0)	21 (7.0)	15 (4.2)	13 (4.4)
Manual occupation	149 (47.8)	149 (49.3)	161 (45.2)	139 (49.1)
Business owner	117 (37.5)	101 (33.4)	120 (33.7)	139 (35.3)
Professional	21 (6.7)	31 (10.2)	60 (16.9)	31 (11.0)
Ownership of mobile phone				
Own mobile phone	257 (82.4)	286 (94.7)	343 (96.3)	274 (96.8)
Main water source (at time of survey)				
Public tapstand	118 (38.6)	38 (12.9)	18 (5.6)	164 (58.6)
Owned private tap	0 (0)	32 (10.9)	40 (12.5)	8 (2.9)
Other private tap	22 (7.2)	98 (33.3)	48 (15)	36 (12.9)
Tank	1 (0.3)	6 (2)	62 (19.3)	2 (0.7)
Kiosk (Sebeya)	0 (0)	0 (0)	4 (1.2)	2 (0.7)
Delivery	0 (0)	0 (0)	0 (0)	1 (.4)
Well	11 (3.6)	2 (0.7)	0 (0)	0 (0)
Shallow well (Bizola)	4 (1.3)	29 (9.9)	0 (0)	0 (0)
Unprotected spring	111 (36.3)	41 (13.9)	0 (0)	0 (0)
Rainwater	39 (12.7)	48 (16.3)	137 (42.7)	67 (23.9)
Surface water	0 (0)	0 (0)	12 (3.7)	0 (0)
Distance to main water source (one-way trip)				
On plot or <5 min	154 (49)	217 (70.2)	280 (78.7)	199 (70.3)
5–15 min	47 (15)	45 (14.6)	57 (16)	67 (23.7)
15–30 min	50 (15.9)	33 (10.7)	17 (4.8)	14 (4.9)
30+ min	63 (20.1)	14 (4.5)	2 (0.6)	3 (1.1)

**Table 3:** Water collection outcomes in Goma.

	Comparison		Intervention			
	*n* (%)		*n*%		Adj OR (95 CI) *	*p*-value
Households collecting water during previous day	246 (63.4)		232 (81.12)		2.53 (1.76 to 3.65)	<0.001
	*n*	Mean (SD)	*n*	Mean (SD)	Adj coef. (95 CI) *	*p*-value
Litres per capita day (lpcd) collected among all households	356	13.53 (15.48)	283	16.33 (14.03)	3.19 (0.84 to 5.53)	0.008
Lpcd collected among households collecting water	246	19.58 (15.1)	232	19.92 (12.98)	0.88 (−1.66 to 3.43)	>0.1
Number of sources usually accessed	356	1.99 (0.55)	283	2.15 (0.63)	0.15 (0.06 to 0.24)	<0.001
Proportion of water collected from primary source during previous day (%)	246	92.83 (14.89)	232	90.74 (16.1)	−1.85 (−4.67 to 0.97)	>0.1
Time per capita day spent collecting water during previous day (minutes)	246	13.08 (17.27)	232	17.78 (16.68)	4.46 (1.98 to 6.93)	<0.001
Amount collected per minute spent collecting (litres)	246	3.08 (1.91)	232	2.06 (1.63)	−0.93 (−1.17 to −0.68)	<0.001
Amount collected per minute spent collecting from tapstands (litres)	15	0.69 (0.3)	156	1.2 (0.82)	0.08 (−0.3 to 0.47)	>0.1
Cost per litre (CDF)	246	4.19 (7.7)	232	4.41 (6.31)	0.29 (−0.95 to 1.53)	>0.1
Cost per litre from improved source (CDF)	100	5.7 (3.0)	191	4.98 (0.82)	−0.61 (−1.09 to −0.13)	0.014

* Adjusted for socioeconomic status and travel-time to primary water source.

**Table 4:** Water use outcomes in Goma.

	Comparison		Full Intervention			
	*n*	Mean (SD)	*n*	Mean (SD)	adj coef. (95 CI) *	*p* Value
Litres per capita day (lpcd) total used	356	9.44 (7.86)	283	8.2 (6.82)	−1.11 (−2.29 to 0.07)	0.066
Lpcd used for bathing	356	2.17 (2.86)	283	1.86 (1.97)	−0.3 (−0.7 to 0.1)	>0.1
Lpcd used for cleaning	356	0.84 (1.37)	283	0.55 (1.11)	−0.3 (−0.5 to −0.1)	0.003
Lpcd used for cooking	356	1.17 (1.44)	283	1.25 (1.61)	0.07 (−0.17 to 0.31)	>0.1
Lpcd used for drinking	356	0.65 (1.68)	283	0.47 (0.82)	−0.19 (−0.41 to 0.02)	0.08
Lpcd used for laundry	356	2.88 (4.71)	283	2.26 (3.6)	−0.48 (−1.16 to 0.19)	>0.1
Lpcd used for sanitation	356	0.81 (1.9)	283	0.67 (1.52)	−0.1 (−0.38 to 0.18)	>0.1
Lpcd used for handwashing	356	0.9 (1.88)	283	0.88 (2.29)	−0.02 (−0.35 to 0.31)	>0.1

* Adjusted for socioeconomic status and distance to main water source.

**Table 5:** Water use outcomes in Bukavu.

	Comparison		BCC Intervention			
	*n*	Mean (SD)	*n*	Mean (SD)	adj Coef. (95 CI) *	*p* Value
Litres per capita day (lpcd) total used	312	5.28 (4.66)	302	5.92 (5.89)	0.68 (−0.31 to 1.66)	>0.1
Lpcd used for bathing	312	1.41 (1.75)	302	1.4 (1.91)	0.06 (−0.28 to 0.4)	>0.1
Lpcd used for cleaning	312	0.57 (0.96)	302	0.72 (1.24)	0.16 (−0.05 to 0.36)	>0.1
Lpcd used for cooking	312	0.89 (1.12)	302	0.94 (1.4)	0.08 (−0.16 to 0.31)	>0.1
Lpcd used for drinking	312	0.29 (0.45)	302	0.34 (0.7)	0.05 (−0.06 to 0.16)	>0.1
Lpcd used for laundry	312	1.56 (2.02)	302	1.65 (2.4)	0.08 (−0.34 to 0.49)	>0.1
Lpcd used for sanitation	312	0.3 (0.8)	302	0.48 (0.89)	0.12 (−0.04 to 0.28)	>0.1
Lpcd used for handwashing	312	0.26 (0.58)	302	0.33 (0.9)	0.09 (−0.05 to 0.23)	>0.1

* Adjusted for socioeconomic status and distance to main water source.

## Data Availability

Anonymised data may be made available upon request. Requests should be directed to Tomas Mosquera (tmosquera@mercycorps.org) or Lamine Keita (lkeita@mercycorps.org).
